# Concomitant Ulecranon Fracture, Ipsilateral Segmental Humerus Fracture and Intercondylar Humerus Fracture in a 4-Year-Old Girl: an Extremely Rare Case Report and Literature Review

**DOI:** 10.5812/atr.8633

**Published:** 2013-06-01

**Authors:** Mohammad Hossein Ebrahimzadeh, Ali Birjandinejad, Ali Sahebjami, Mohammad Hossein Taraz Jamshidi

**Affiliations:** 1 Orthopedic and Trauma Research Center, Mashhad University of Medical Sciences, Mashhad, IR Iran

**Keywords:** Elbow Fractures, Upper Extremity, Humerus Fracture, Pediatric Trauma, Iran

## Abstract

T-condylar fracture of distal humerus in young children is very rare. Pure physeal fractures of the olecranon are also rare. We report on an extremely rare case of concomitant ulecranon fracture, ipsilateral segmental humerus and intercondylar humerus fracture (type III) in a skeletally immature patient.

## 1. Introduction

T-condylar fracture of distal humerus in young children is very rare (1-4). Pure physeal fractures of the olecranon are also rare (2). We report a rare case of concomitant ulecranon fracture, ipsilateral segmental humerus and intercondylar humerus fracture (type III) in a skeletally immature patient. The incidence, classifications, mechanism and management of this unusual injury are discussed.

## 2. Case Report

**Figure 1.**
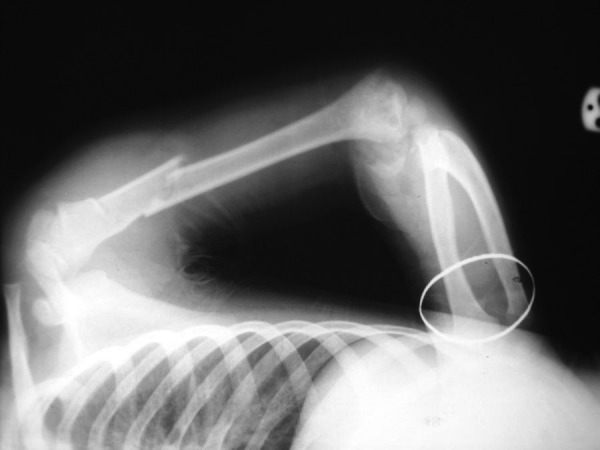
Initial X-Ray of the Fracture

**Figure 2.**
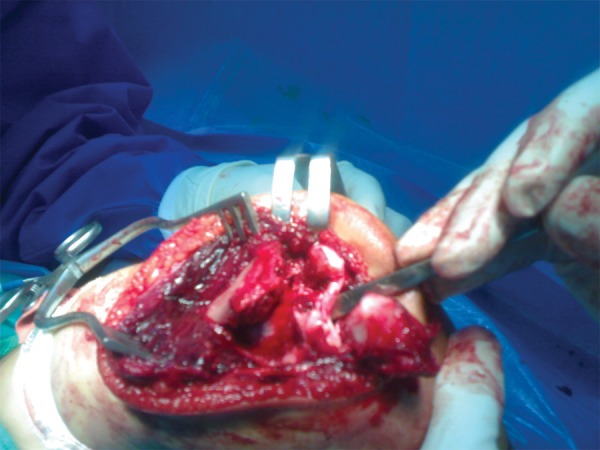
Photography During Surgery

**Figure 3.**
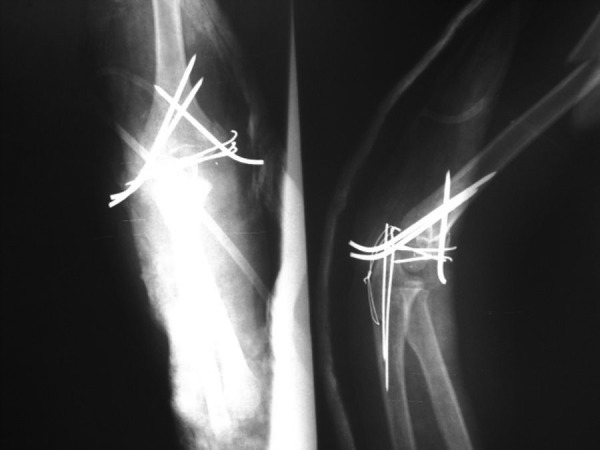
AP and Lat X-Rays After Open Reduction and Internal Fixation of the Fractures

**Figure 4.**
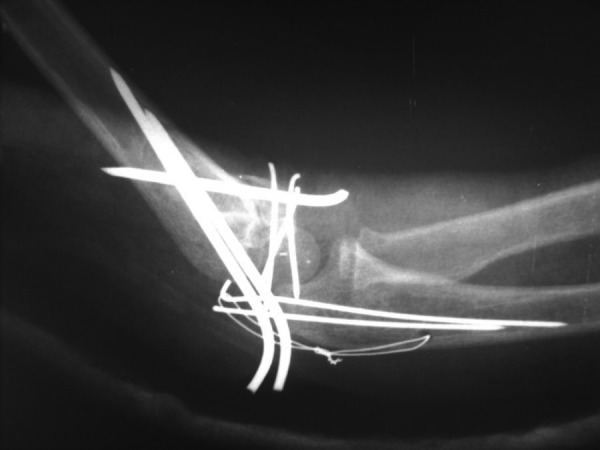
Control X-Ray After 3 Weeks Postoperative

A 4-year-old girl with multiple traumas due to a car accident was admitted to the emergency department of our hospital 4 hours after injury. Her vital signs were stable. Her right elbow was grossly swollen and bruised with semi extended position and a laceration 0.5 × 0.5 cm in medial proximal humerus. Abnormal mobility and crepitus were felt at the extremity. Anteroposterior radiography showed a fracture of the proximal diaphysis of the humerus (type IIIA) and probably a supracondylar fracture of the distal humerus (Figure 1). In the emergency operating room prophylactic antibiotic was given and she was placed in the lateral position. Under general anesthesia the wound was irrigated with sterile isotonic saline, then scrubbed and the arm was prepared and draped. Laceration was irrigated and debrided. Elevated for 2 minutes and exsanguinated by a cotton elastic bandage. A Pneumatic tourniquet was applied. With a long posterolateral approach, dissection was deepened through the fascia; aponeurosis of the triceps was exposed as far distally as its insertion on the olecranon. Then the remaining muscle fibers were incised in the midline. The periosteum was elevated together with the triceps muscle from the posterior surface of the distal humerus for 4 cm. For wider exposure, we continued the subperiosteal stripping on each side as conservatively as possible so that serious damage to the blood supply of the bone would be avoided by releasing the muscular and capsular attachments to the medial condyle. The ulnar nerve was exposed. During the operation we noticed the patient had both a T-condylar and an olecranon fracture,the latter had been broken but not displaced because of thick articular cartilage component and intact periosteum (Figure 2). Our first priority was to reestablish the integrity of the articular fragments, in other words, to convert it into a supracondylar fracture with 3 pins from medial condyle. The olecranon and coronoid fossae were cleared from bony fragments or debris to eliminate the chance of bony impingement. When the condylar and articular integrity was reestablished, the distal fragments were secured to the proximal fragment by stabilizing the supracondylar fragment columns. We reduced the fracture by applying longitudinal traction and manipulation. The elbow was flexed to neutral position to cross the two smooth Steinmann pins through the condyles and metaphys. The olecranon had been exposed previously but was not dissected from the soft tissue. While an assistant grasped the olecranon with a towel clip, the fragments were reduced and fixed with two parallel intramedullary Kirschner wires, a stainless steel wire through a transverse hole was drilled in 4 cm of the distal fragment, a figure of eight tension bands were then applied. The reflected aponeurosis of the triceps was reattached. The radial pulse was checked. After a relative reduction of proximal segmental humerus fracture, an above elbow plaster of Paris slab with sling and swathe was applied post-operatively from shoulder to MCP (Figure 3). We followed the patient closely for one year (Figure 4); after 3 months post-surgery we removed all pins and wires and there were no infection, no neurologic deficit and at six months, the patient achieved a full range of motion.****

## 3. Discussion 

The actual incidence of T-condylar fractures in younger children is certainly rare, but it may be under diagnosed (5-10). Special imaging studies such as arthrograms are necessary to demonstrate an intracondylar fracture (2), because it is often confused with other fractures, such as those involving the lateral condylar physis or total distal physis (1, 5, 9). The use of CT and MRI in acute injuries does not appear to have much practical value. In this case, initially we hadn't diagnosed an intercondylar fracture either. Fracture of the olecranon epiphysis is the rarest form of epiphyseal detachment (2, 11). Only 16 acute fractures in which apiphyseal involvement is mentioned can be found in the literature (4, 11-13). In the French literature, Bracq described 10 patients in whom the fracture was distal and parallel to the apophyseal line and then crossed it at the articular surface (14). In T-condylar fractures, the fracture line originates in the central groove of the trochlea and courses proximal to the olecranon and the coronoid fossae, where it divides and separates the medial and lateral bony columns of the distal humerus (2). In our case, fracture line originated between the trochlea and capitellum with a free piece of lateral trochlea in the joint. Various classifications (15-17) for adult T-condylar fractures have been proposed, but there are problems with applying these classifications to children's injuries. For example, the number of children with this fracture is so small that no clinician can encounter all types of fracture patterns during his or her own experience (2). Toniolo and Wilkins have classified T-condylar fracture; three major types have been identified based on the degree of displacement and comminution of the fracture fragments. Type I fractures are minimally displaced, type II fractures are displaced but do not have comminution of the metaphyseal fragments, type III fractures are displaced fractures with comminution of the metaphyseal in particular surface and lateral condyl. Papavasiliou, Beslikas and Nenopoulos classified isolated fractures of the olecranon in children as intra articular (group A) and extra articular (group B). Included in the intra articular fracture are simple crack fractures, fractures with minimal displacement, complete fracture of the olecranon involving the articular cartilage with slight dorsal displacement of the proximal fragment and grossly displaced fracture (2, 18). In our case, there was pure apophyseal avulsion without displacement that attributes to the thicker articular cartilage and intact periosteum in younger children, while in older children those fractures tend to be displaced. The most common mechanism producing intercondylar humerus fractures is a direct blow to the posterior aspect of the elbow (19). In these flexion injuries, the wedge effect is produced at the apex of the trochlea by the central portion of the trochlear notch (2). In flexion injuries, the condylar fragments usually lie anterior to the distal shaft. Injuries to the apophysis of the olecranon is due to avulsion forces across the apophysis occurring with the elbow flexed, similar to the more common flexion metaphyseal injuries (2). In the present case, however, the injury occurred after a high-energy trauma. Segmental humerus fracture is due to compression, bending and torsional force. Concomitant olecranon fracture and ipsilateral segmental humerus and intercondylar humerus fracture in a skeletally immature patient is an exceptional injury. The mechanism of injury probably involves a direct blow to the posterior aspect of the elbow in flexion posture so that the wedge effect of the central portion of the trochlear notch produces T-condylar fracture and olecranon fracture at the level of coronoid, on the other hand, continuing of the compression force with proximal humerus hinge in combination with bending and torsional force produces a segmental humerus fracture. Because of the rarity of T-condylar fractures in children, there is no standard recommended treatment (1, 2, 15, 18, 20-22). Closed methods alone usually can't produce an acceptable result for T-condylar fracture because of instability (for example, the muscle forces applied to the fragment) (2). Zimmerman advocated establishing an anatomic reduction with internal fixation so that early motion could facilitate a more rapid rehabilitation (5, 9). In two cases in young children described by Beghin and colleagues, operative intervention was necessary to achieve a satisfactory reduction (1). An anatomical reduction with internal fixation causing early motion that can facilitate a more rapid rehabilitation (22, 23) . Therefore, our first consideration regarding these fractures is to reestablish the integrity of the articular surface to maintain the congruity of the joint. During the operation we noticed that the olecranon had been broken but not displaced because of the thick articular cartilage component and intact periosteum. This evidence helped us to see the articular joint better. For stability we made a good integrity for the lateral and medial supracondylar columns. For the most rigid biomechanical construct, 3 pins were used and the pins were crossed several centimeters proximal to the humerus and not at the fracture site. For elbow articular mobility we reconstructed articular congruity, corrected alignment of the axis of motion, freed fossae from debris and bone. In our case, accurate anatomic reduction of the articular surface of the distal humerus and rigid fixation to the diaphysis allowed the patient to undergo early physiotherapy.
